# A case of left atrial thrombus detected by intra-left atrial echocardiography

**DOI:** 10.1016/j.hrcr.2024.11.006

**Published:** 2024-11-12

**Authors:** Takuo Tsurugi, Ken Okumura, Yoko Horibata, Tomohiro Sakamoto, Junjiroh Koyama

**Affiliations:** Saiseikai Kumamoto Hospital, Cardiovascular Center, Kumamoto, Japan

**Keywords:** Left atrial thrombus, Intra-left atrial echocardiography, Atrial fibrillation, Catheter ablation, Maze operation

**Key Teaching Points**

• Routine intra-left atrial echocardiography was critically useful in detecting posterior wall thrombus in the left atrium, which was underestimated by transesophageal echocardiography before the ablation procedure for atrial fibrillation.
• In the case of a maze procedure and removal of a left atrial appendage, a posterior wall in the left atrium is one of the main targets to be observed by transesophageal echocardiography, in addition to guideline-directed standard views to rule out the presence of thrombus.
• It is essential to recognize the advantage and limitation of each modality, including transthoracic echocardiography, transesophageal echocardiography, contrast-enhanced cardiac computed tomography, and intracardiac echocardiography in evaluating thrombus in the left atrium, and choose them properly, according to the risk and history of each patient with atrial fibrillation.

## Introduction

Pulmonary vein isolation (PVI) is a cornerstone of atrial fibrillation (AF) ablation. Because the PVI procedure needs a complex catheter manipulation in the left atrium (LA), it is accompanied by relevant complications.^1^^,^^2^ The recent Japanese Catheter Ablation registry showed a 3.2% incidence of complications, including major bleeding (1.2%), cardiac tamponade (0.7%), systemic embolism (0.2%), phrenic nerve paralysis (0.6%), and gastrointestinal insufficiency (0.2%), occurring in 40,000 AF ablations in 2018.^3^ Although the incidence of systemic embolism, especially cerebral infarction, is low, we have to keep it in mind to minimize or eliminate the risk of procedure-related embolism by an appropriate periprocedural anticoagulation, including oral anticoagulant administration before and after the procedure and appropriate heparinization during the procedure, and preprocedural assessment of the presence or absence of LA thrombus.^1^^,^^2^ For the assessment of LA thrombus, transesophageal echocardiography (TEE) is recognized as a gold standard^4^ and cardiac computed tomography (CCT) with contrast medium enhancement is an alternative to TEE.^5^ We recently reported the usefulness of intracardiac echocardiography (ICE) from the LA cavity (LA ICE) in real-time visualization of the esophagus during the procedure and assessment of the LA posterior wall thickness and its relation to the esophagus.^6^ Herein, we present the case of LA posterior wall thrombus, which was underestimated by TEE done 9 days before the ablation procedure and was clearly demonstrated by LA ICE before inserting the ablation catheter into the LA cavity.

## Case report

**Figure 1:**
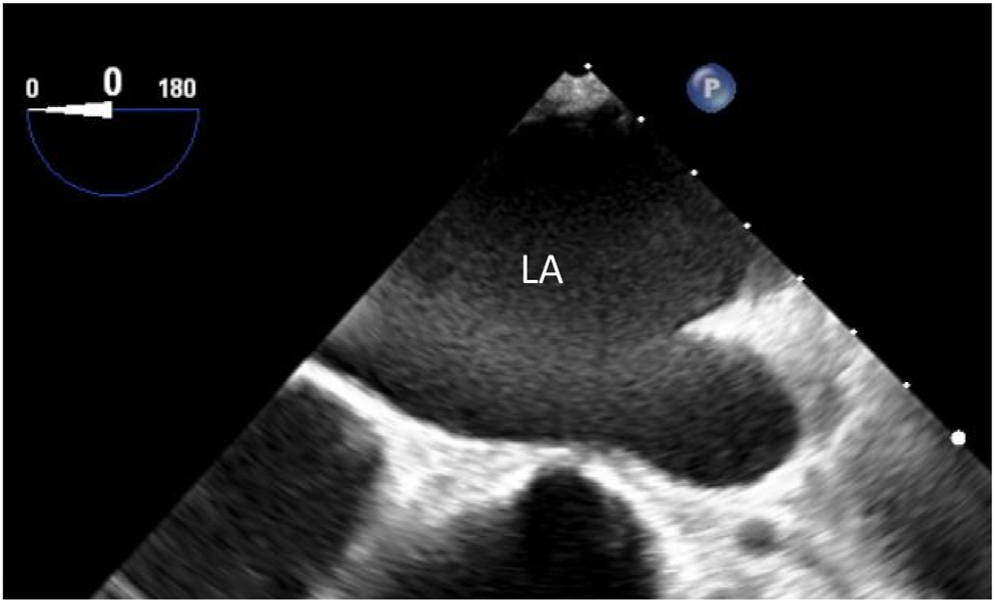
Transesophageal echocardiography performed 9 days before catheter ablation for atrial fibrillation. Spontaneous echocardiographic contrast was documented. LA = left atrium.

The patient was a 55-year-old man with dyspnea on effort and diagnosed with moderate mitral regurgitation and paroxysmal AF. Mitral valvuloplasty was conducted for mitral valve prolapse at the medial scallop of the posterior leaflet. In the operative findings, tendon cord rupture was documented and no remarkable rheumatic degeneration was observed. Suture of elongated valve leaflets and ring annuloplasty was undertaken. Because he also had symptomatic paroxysmal AF, an LA maze procedure and LA appendage removal and suture were also performed. Six years after the surgery, he developed recurrent paroxysmal AF and sick sinus syndrome, resulting in implantation of a dual-chamber pacemaker and administration of 60 mg of edoxaban as anticoagulation therapy. His CHA_2_D_2_-VASc score was 2. Unfortunately, he had discontinued taking edoxaban 3 months after the pacemaker implantation. In addition, 5 months later, he suddenly became aware of numbness in his face and right upper and lower extremities, which disappeared within 24 hours and was diagnosed as transient ischemic attack. During this hospitalization, head magnetic resonance imaging, cervical vascular echocardiography, and transthoracic echocardiography were performed; none of which revealed obvious abnormal findings related to transient ischemic attack. From a retrospective viewpoint, a thrombus was indicated in the LA by TTE; however, halation of the mitral valve ring obscured an accurate diagnosis. Unfortunately, no TEE was performed at that time. Therefore, edoxaban 60 mg was readministered and he was discharged from the hospital. Because repetitive episodes of AF had been documented on the pacemaker records, we planned to perform a catheter ablation for recurrent AF after confirming that he took edoxaban stably for 3 months. TTE performed 9 days before admission for the ablation showed dilatated LA (52 mm), isolated basal hypertrophy (interventricular septum 14 mm and posterior wall 10 mm), and mild mitral regurgitation. Spontaneous echocardiographic contrast and small diverticulum (7 mm) in the left anterior atrium were documented with TEE, but no definite thrombus was detected (Figure 1). CCT was performed without use of contrast medium for his impaired renal function.

**Figure 2:**
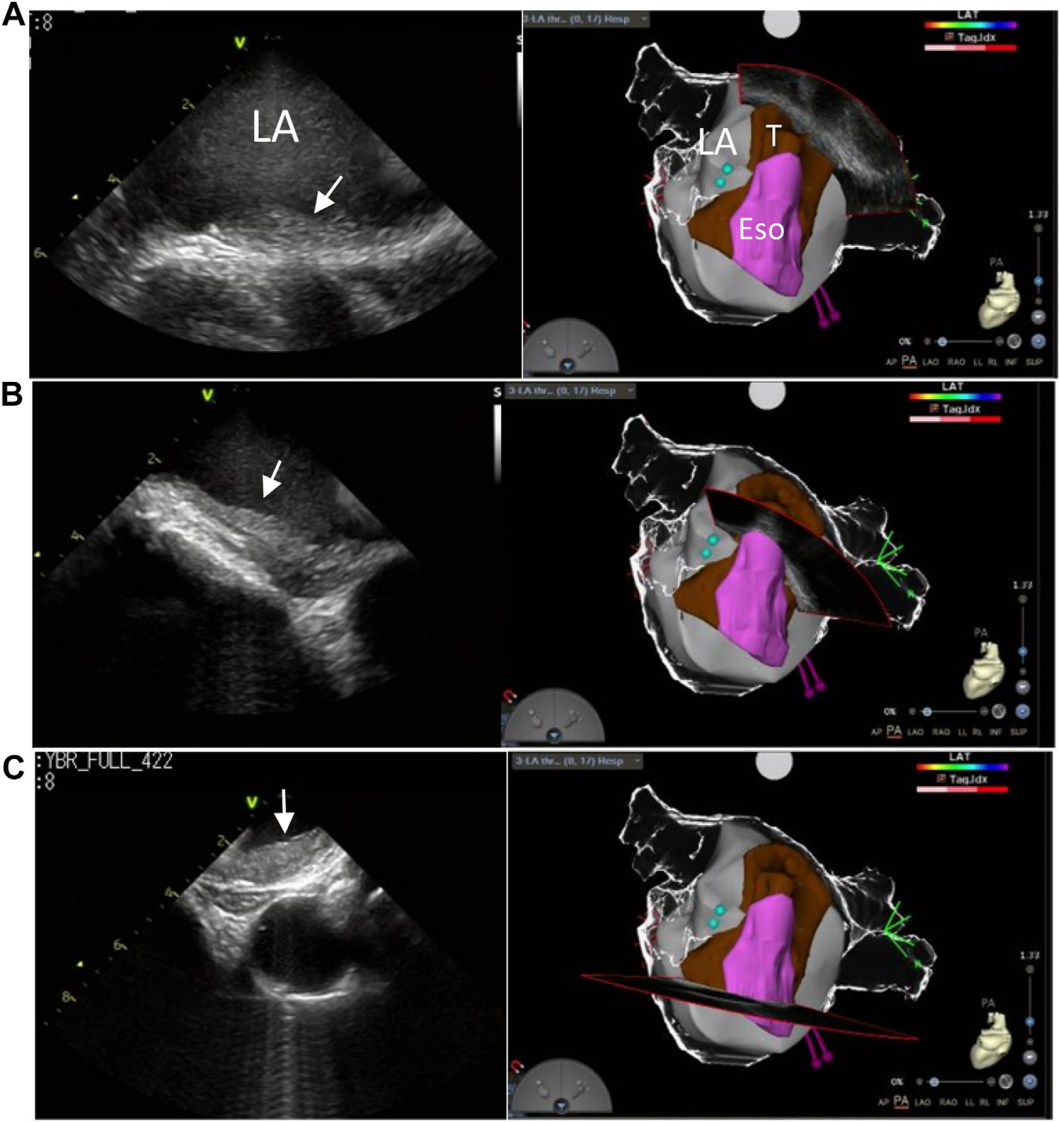
Intracardiac echocardiography from the left atrial (LA) cavity demonstrated that the hyperechoic and flat mass lesion adhered to the posterior wall (*arrows*). Transverse views at high (**A**), middle (**B**), and low levels (**C**) of the LA posterior wall are shown. LA posterior views of CARTO (Biosense Webster, Diamond Bar, CA) image are provided in the *right*. *Brown color* indicates thrombus and *pink color* indicates esophagus.

**Figure 3:**
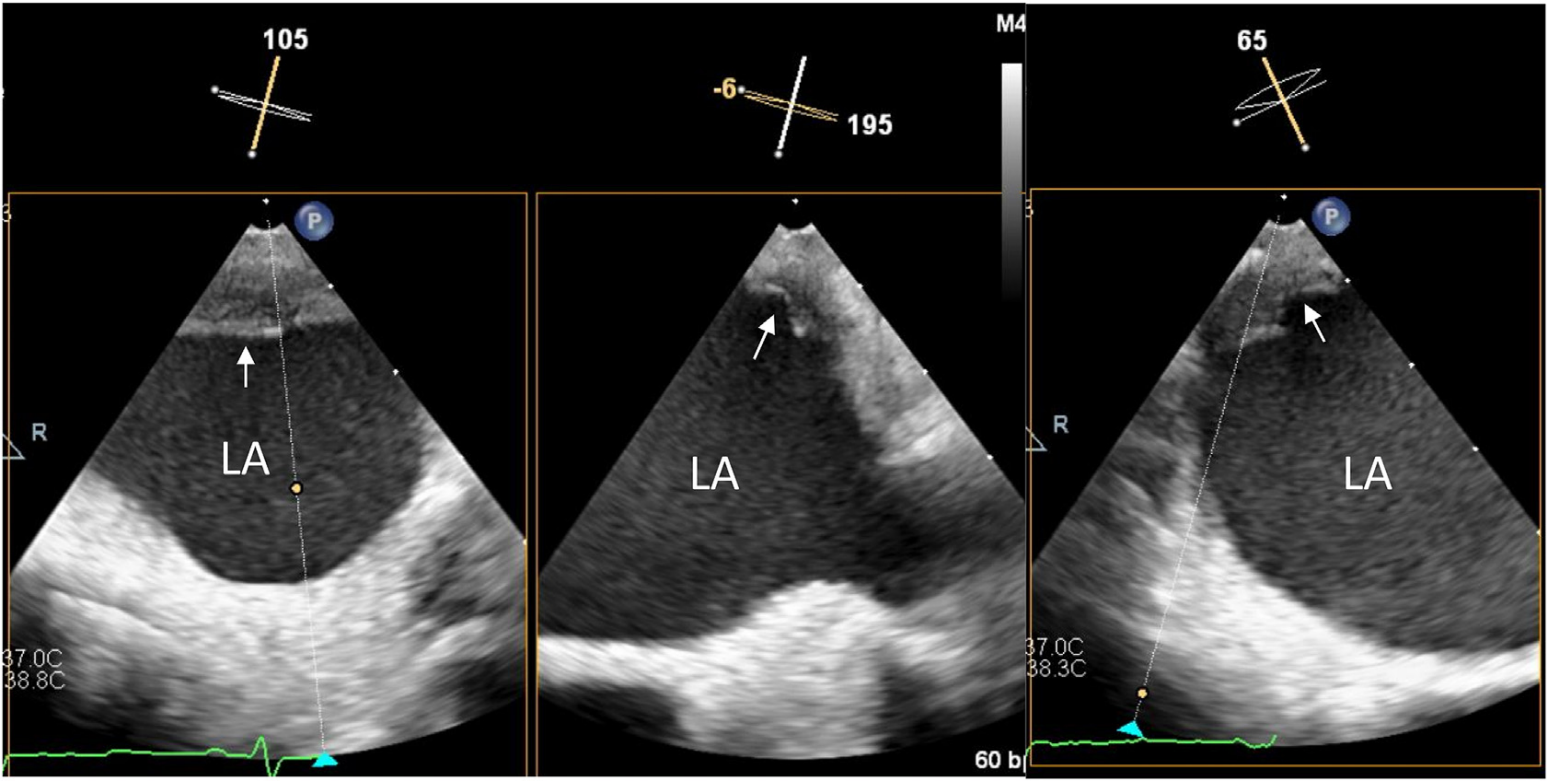
Transesophageal echocardiography done just after termination of the procedure. The posterior wall thrombus was documented in the views not directed by guideline (*arrows*).

In our institution, LA ICE is performed routinely in every patient undergoing AF ablation. After anticoagulation with 3000 international units (IU) of heparin injected before and 5000 IU just after the transseptal puncture, an 8F CARTO SOUNDSTAR® ultrasound catheter (Biosense Webster, Diamond Bar, CA) was inserted into the LA cavity through an 8.5F long sheath (SL0; St Jude Medical, St Paul, MN). As described previously, the LA ICE image was created digitally with the use of a Vivid™ iq imaging console (GE Healthcare, Fairfield, CT).^6^ During observation with LA ICE, we noticed the hyperechoic mass lesion adhered over the LA posterior wall (Figure 2). We first thought it was an artifact of echo signals. However, it was not changed by other angles and the lesion was continuously visualized from the LA roof to the inferior posterior wall. We diagnosed this hyperechoic lesion as atrial thrombus because it showed homogeneous echo density and spread flat over the LA posterior wall. Then we terminated the procedure by removing all catheters and sheaths, and performed TEE. We reconfirmed the thrombus covered almost all of the LA posterior wall (Figure 3). Reassessment of TEE done before ablation revealed that the abnormal echo suggesting thrombus was vaguely shown in some views. Immediately after the procedure, we administered 10,000 IU/d of heparin by continuous intravenous infusion and adjusted the dose to achieve an activated partial thromboplastin time of 60–100 seconds by blood sampling twice per day. On the same day, warfarin was administered instead of edoxaban. Six days after the procedure, prothrombin time with international normalized ratio of 2.38 was reached and heparin was discontinued. The patient did not show any neurologic abnormalities after the procedure. We followed the patient every 3 months with TEE, and almost all thrombus disappeared 1 year later.

## Discussion

With the use of LA ICE done before the PVI procedure, we detected a large amount of thrombus in the LA posterior wall. We terminated all the following procedures without any adverse events. In patients with low CHA_2_D_2_-VASc score and paroxysmal AF, the evaluation of LA/LA appendage may be skipped when the patients have been effectively anticoagulated for a certain period. In the present case, the patient had been properly anticoagulated for approximately 3 months before the ablation procedure, but had the LA thrombus. This patient seemed to be at high risk of thrombus formation because of his history of LA maze procedure and transient ischemic attack and spontaneous echo detected by TEE.

There are 2 reasons for the difficulty in diagnosis of the LA thrombus by TEE in this case. First, it is rare for thrombus to be formed over the LA posterior wall. In evaluating thrombus with TEE before AF ablation, >90% are documented in the LA appendage,^7^ and the detection rate was reported to be approximately 1% to 6%, which varies, depending on the patient's CHADS_2_ or CHA_2_D_2_-VASc scores, paroxysmal or nonparoxysmal AF, treatment with vitamin K antagonist or direct oral anticoagulants, and undergoing cardioversion or catheter ablation.^8^ Rheumatic mitral stenosis is one of the common causes of LA thrombosis. Kaymaz et al^9^ reported that 474 cases of intraoperative assessment for thrombi in patients with rheumatic mitral valve disease, thrombi were identified intraoperatively in 105 cases, 90 in the LA appendage and 15 in the LA, 12 of which were in the posterior wall. In patients with the LA appendage surgically removed, there are few data about the prevalence of LA thrombus. Taminishi et al^10^ reported a case in which a large hyperechoic mass adhering to the LA posterior wall was detected by TEE 2 years after a maze procedure.^10^ Because the thrombus was not diminished with anticoagulation therapy, surgical resection was performed and the thrombus was found to originate on the ablation line made by the maze procedure. Radiation therapy following esophageal cancer surgery^11^ or posterior wall isolation by radiofrequency ablation in a case with very low ejection fraction^12^ were reported to be associated with formation of thrombus in the LA posterior wall.

The configuration of thrombus was flat and expanded almost whole in the posterior wall. Structures in the near field from a probe, such as a TEE probe close to the LA posterior wall, are sometimes obscure due to the high amplitude of oscillations by the transducer itself, causing so-called “near field clutter.”^9^ The mass lesion, which was not protruded as seen in this case, might be difficult to distinguish thrombus from an ultrasound image artifact. In a routine preoperative evaluation, the LA appendage is the main focus of investigation. In the present case, the LA appendage was removed, LA maze operation was done, and spontaneous echo was present. All of these indicate that the posterior wall should have been observed more carefully, in addition to guideline-directed standard views, to rule out the presence of thrombus.

LA ICE was very useful to diagnose homogeneously overlaid thrombus in this case. In general, for LA appendage observation with ICE, the view from the right ventricular outflow tract, pulmonary artery, or left superior pulmonary vein was available. Even though the LA body could be observed from the right atrium, the direct observation from the LA was recommended for precise evaluation especially for the LA posterior wall. As we described previously, LA ICE provided important information on the location of the esophagus, LA wall thickness, and the relation of LA posterior wall to the esophagus.^6^ Although TEE showed very high sensitivity (93%) and specificity (100%),^13^ the ActionICE II study reported that ICE pointed out a false-positive rate of 9 of 21 (43%) in the thrombus diagnosed by TEE, and suggested a valuable option for verification of a TEE-based diagnosis of a thrombus.^14^

Recently, contrast-enhanced CCT in the late phase was shown as a reliable alternative to TEE. A meta-analysis reported that the accuracy for the diagnosis of thrombus compared with TEE was 95% for sensitivity and 89% for specificity in early imaging studies, and 98% for sensitivity and 100% for specificity for delayed imaging studies.^15^ However, there are several limitations for CCT and Romero et al^5^ reported the positive predictive value ranging from 41% to 92%, poor interobserver variability, and difficulties in hemodynamic and functional assessment. Requirement for contrast medium also limits the indication of CCT for patients with contrast medium hypersensitivity or impaired renal function, as in this case.

## Conclusion

A case of the LA posterior wall thrombus detected by LA ICE was reported. We should recognize the advantage and limitation of each modality in evaluating thrombus in the LA, and choose them properly according to the risk, especially the maze procedure and mitral annuloplasty in this case, and history of each patient.

## Disclosures

The authors have no conflicts of interest to disclose.
